# The Hypoxia‐Associated High‐Risk Cell Subpopulation Distinctly Enhances the Progression of Glioma

**DOI:** 10.1002/advs.202416231

**Published:** 2025-03-06

**Authors:** Quan Wan, Xuechao Wu, Jinxu Zhou, Weiqi Wu, Yuanliang Cao, Cuiping Sun, Zheng Li, Zhicheng Gong, Hong Tang, Qilin Li, Junsheng Chu, Qing Wang, Kaisa Cui, Xiaojie Lu

**Affiliations:** ^1^ Department of Neurosurgery and Emergency Medicine Jiangnan University Medical Center (Wuxi No.2 People's Hospital) Wuxi Jiangsu 214002 China; ^2^ Neuroscience Center Wuxi School of Medicine Jiangnan University Wuxi Jiangsu 214122 China; ^3^ Wuxi Neurosurgical Institute Wuxi Jiangsu 214043 China; ^4^ Department of Neurosurgery The 904th Hospital of Joint Logistic Support Force of PLA Wuxi Jiangsu 214044 China; ^5^ Department of Neurosurgery Beijing Tiantan Hospital Capital Medical University Beijing 100070 China; ^6^ Wuxi Cancer Institute Affiliated Hospital of Jiangnan University Wuxi Jiangsu 214062 China; ^7^ Department of Pathology Jiangnan University Medical Center (Wuxi No.2 People's Hospital) Wuxi Jiangsu 214002 China; ^8^ Computer Vision Lab Department of Electrical Engineering California Institute of Technology Pasadena California 91125 USA

**Keywords:** glioblastoma, hypoxia, lower‐grade glioma, organoid, single‐cell sequencing

## Abstract

Less‐aggressive lower‐grade gliomas (LGGs) frequently transform into glioblastoma (GBM). Most previous studies of gliomas have not focused on LGG‐original high‐risk subpopulations, which may be one of the most critical hallmarks of glioma progression. In this study, LGG samples are collected to perform single‐cell sequencing (scRNA‐seq) and identify a unique cell subpopulation marked by CDC20, KIF20A and PTTG1, correlating with poor survival in multiple cohorts. Importantly, the CDC20^+^KIF20A^+^PTTG1^+^ cell subpopulation is strongly associated with transforming LGG to GBM according to scRNA‐seq and multiplexed immunofluorescence staining assays. In vitro, ex vivo and in vivo investigations further hint that this cell subpopulation is critical to the proliferation and growth of gliomas, and is associated with the hypoxia core activation. Pharmaceutically and therapeutically, the inhibition of this cell subpopulation showed significant anti‐tumor effects and effective enhancement of the Temozolomide treatment efficiency. These findings provide insights into the therapeutic strategies of glioma progression, highlighting promising ways to avoid early‐stage gliomas developing into advanced gliomas.

## Graphical abstract

This study suggests the potential roles of a novel hypoxia‐associated CDC20^+^KIF20A^+^PTTG1^+^ cell subpopulation in glioma progression. This high‐risk glioma cell subpopulation is therapeutically vulnerable to glioma progression and may play important roles in glioma standard‐of‐care therapeutic resistance. These findings highlight a promising strategy to impede the progression of early‐stage glioma to advanced disease by targeting this subpopulation.

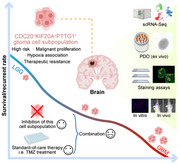

## 1 Introduction

Although lower‐grade gliomas (LGGs) are less aggressive than high‐grade gliomas in terms of neuron imaging and histopathological features, many patients die from tumor progression and malignant transformation. Tumor resection is one of the most important factors affecting overall survival, progression‐free survival and malignant transformation.^[^ ^1^ ^]^ However, complete neurosurgical resection is impossible due to the highly invasive nature of gliomas and the loss of brain function caused by radical surgery.^[^ ^2^ ^]^ Surgery or chemotherapy‐mediated cell subtypes may play critical roles in tumor progression, such as progression to glioblastoma (GBM, a WHO grade IV glioma type), while other subsets may remain stable for years.^[^ ^2^ ^]^ Thus, it's urgent to identify and recognize such kind of cell subpopulations and related phenotypic features to utilize appropriate chemotherapies to restrain tumor progression.^[^ ^3^ ^]^

Single‐cell RNA sequencing (scRNA‐seq) reveals the tumor composition and genetic expression features of tumor cells with distinct phenotypes and epigenetic states. The study by Alan T Yeo et al. provided insight into the changes in the immune microenvironment during the progression of GBM,^[^ ^4^ ^]^ Ekaterina Friebel et al. revealed the heterogeneous composition of tissue‐resident and aggressive immune cells within the highly immunosuppressive tumor microenvironment (TME).^[^ ^5^ ^]^ However, most previous studies of gliomas haven't focused on LGG and thus limiting our understanding of high‐risk cell subpopulations, which may be one of the most critical hallmarks of the aggressiveness and progression of LGG.

Because of the inability to accurately and faithfully model LGG ex vivo to support preclinical target identification, the standard treatment regimens have not been adjusted for over 15 years despite advances in our collective understanding of glioma pathogenesis and drug sensitivity studies. The increasing application of patient‐derived organoids (PDO) modeling to glioma research provides us with a new approach to recapitulate parental tumor characteristics. Compared to traditional cancer cell lines and mouse models, organoid models maintain diverse cell types, which enables further diverse research, such as studies on the TME. Particularly in basic and translational research on LGG lacks cell lines from primary samples support though widespread efforts have been made.

Tumor hypoxia is linked to the aggressive nature of gliomas, leading to first‐line therapy resistance and recurrence.^[^ ^6^ ^]^ However, hypoxia has remained overlooked in the clinical treatment of gliomas.^[^ ^6^ ^]^ Recent studies using scRNA‐seq, spatial or PDO biotechnology demonstrated that hypoxia plays important roles in the GBM microenvironment, such as glioma cells and myeloid cells.^[^ ^7^, ^8^ ^]^ Herein, tissues from LGG cases were collected for scRNA‐seq examination. We identified a unique CDC20^+^KIF20A^+^PTTG1^+^ high‐risk glioma cell subpopulation probably necessary for glioma progression of LGG/GBM transformation, which was further explored in multiple cohorts and validated in samples by immunohistochemistry and multiplex immunofluorescence assays. Furthermore, our in‐depth analysis and functional experiments in vitro, ex vivo and in vivo suggest a potential association between the hypoxia core activation and this high‐proliferative/growth high‐risk glioma cell subpopulation. Finally, we highlight the therapeutic strategy to decelerate glioma tumor progression and enhance the standard‐of‐care therapy efficiency of glioma by inhibiting this glioma cell subpopulation.

## 2 Results

### 2.1 Identification of High‐Risk Glioma Cell Subgroups in LGG Single‐Cell Atlas

**Figure 1:**
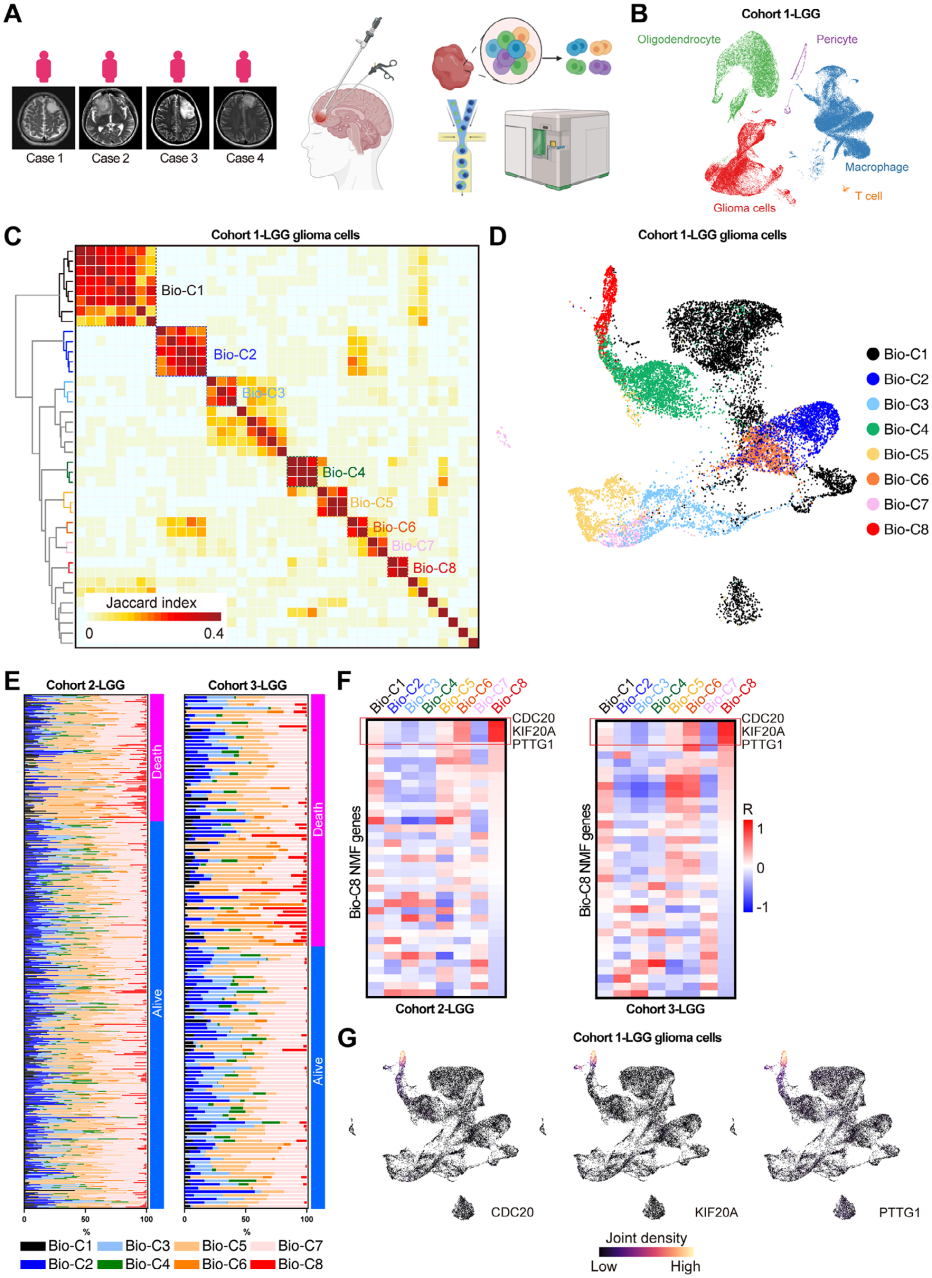
Identification of high‐risk glioma cell subgroups in LGG single‐cell atlas. A) Medical imaging of each case from cohort 1 and single‐cell RNA‐Seq experimental procedure. This panel was created with BioRender.com. B) UMAP plots colored by cell types from cohort 1 of LGGs. C) Heatmap depicting shared intra‐tumor expression programs and eight biological clusters across cohort 1 patients. D) UMAP plots colored by eight biological clusters from the cohort 1. E) Bar plots colored by each biological‐clusters distribution in cohort 2 and 3 of LGGs. F) Heatmaps showing the correlations between Bio‐C8 proportions and top genes in each NMF programs of Bio‐C8. G) UMAP plots colored by expression density of Bio‐C8 specific marker genes CDC20, KIF20A and PTTG1 in the cohort 1.

LGG has highly variable clinical behavior and a subset of these gliomas will progress to GBM within months.^[^ ^1^ ^]^ Thus, we first profiled the scRNA‐seq data of four LGG patients (Cohort 1, **Figure** **1**A,B). Overall, 101682 single‐cell transcriptome cells passed stringent quality filtering steps after alignment and read counting. Cell cluster‐specific genes coalesced clusters into five major groups according to canonical marker expressions  displayed in Figure S1A,B (Supporting Information): glioma cells (GFAP and PTPRZ1), T cells (CD3D and CD3E), oligodendrocyte (MOG), macrophages (CD68) and pericyte (RGS5). Cells from different patients were clustered together in each cell type, indicating that our data were not influenced by batch effects (Figure 1B).^[^ ^25^ ^]^ Glioma cell distributions were relatively uniform among different patients (Figure S1C–E, Supporting Information). Moreover, we adopted InferCNV to predict copy number status with a single‐cell gene expression matrix (Figure S1F, Supporting Information).^[^ ^15^ ^]^ Compared with other cell types, glioma cells exhibit meaningful copy number alterations, such as chr7 gain, chr10 loss and chr19q loss, revealing that these cells are potentially malignant cells.

To explore intra‐tumor expression heterogeneity in the LGG cell population, NMF intra‐tumor expression programs that consist of co‐expressed genes in each tumor were learned and defined. Together, we dissected 40 intra‐tumor expression programs and classified eight biological clusters (Bio‐Custers) shared in Cohort 1 (Figure 1C,D). Next, we predicted the cellular composition of Bio‐C1∼8 in primary LGG bulk RNA‐seq data Cohort 2 and 3 from Cohort 1 LGG single‐cell data using deconvolution with Bayesian integrative analysis.^[^ ^18^ ^]^ Intriguingly, Bio‐C8 was relatively enriched in death patients compared with those who survived (Figure 1E). Additionally, we divided each Bio‐Custer into high‐ and low‐activity groups based on a previously reported method.^[^ ^26^ ^]^ We observed that high Bio‐C8 group was associated with poorer survival in two independent LGG cohorts (Figure S1G,H, Supporting Information). These results suggest that the Bio‐C8 subpopulation is strongly associated with clinical outcomes in patients with LGG. Subsequently, we need to obtain sign genes of the Bio‐C8 subpopulation. The top 100 genes in each NMF program of Bio‐C8 were collected, genes that were also in NMF programs of Bio‐C1∼7 were excluded. The correlation levels of these genes were subsequently calculated for Bio‐C1∼8 proportions. Interestingly, CDC20, KIF20A and PTTG1 were the top three genes in both Cohorts 2 and 3 of LGG (Figure 1F). CDC20, KIF20A and PTTG1 were specifically co‐expressed in Bio‐C8 LGG glioma cells (Figure 1G). Taken together, the Bio‐C8 cell subpopulation is associated with poor survival in LGG, and can be characterized by CDC20, KIF20A and PTTG1 expression.

### 2.2 Stratification Based on Expressions of CDC20, KIF20A and PTTG1 is Associated with LGG Prognosis

**Figure 2:**
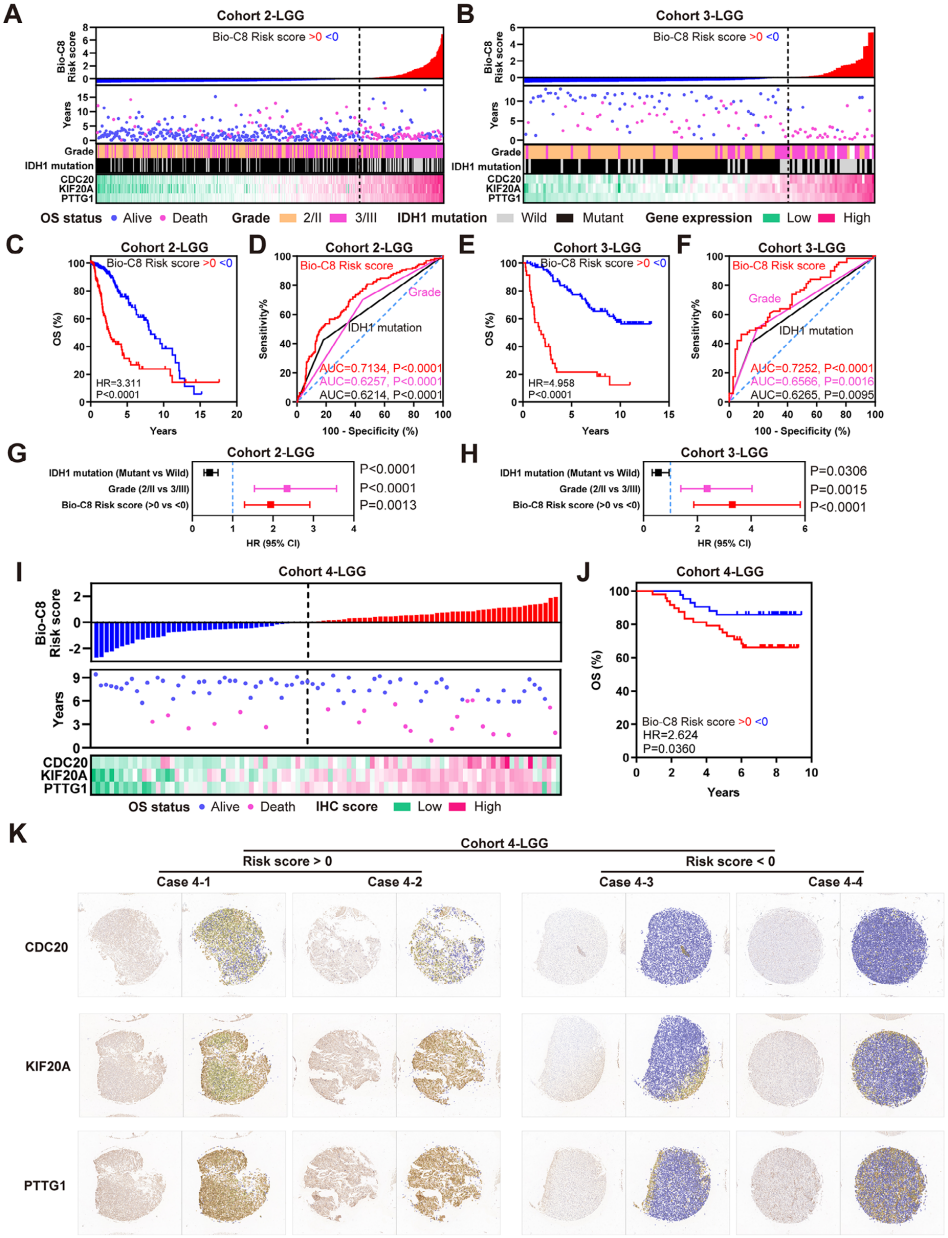
Stratification based on the expressions of CDC20, KIF20A and PTTG1 are associated with LGG prognosis. A,B) Bio‐C8 risk score distribution (top) with patient survival status (middle), grade and IDH mutation status and gene expression (bottom) in cohort 2 (A) and 3 (B). C) Kaplan–Meier curve showing the OS of Bio‐C8 risk score in the cohort 2. D) ROC showing the AUC of Bio‐C8 risk score in the cohort 2. E,F) Similar to (C,D), but in the cohort 3. G,H) Multivariate COX regression models showing the effect to OS of Bio‐C8 risk score, grade and IDH mutation status in cohort 2 (G) and 3 (H). I) Bio‐C8 risk score distribution (top) with patient survival status (middle), and IHC score (bottom) in cohort 4 of LGG samples. J) Kaplan–Meier curve showing the OS of Bio‐C8 risk score in the cohort 4. K) IHC staining images of CDC20, KIF20A and PTTG1 in cohort 4.

To further explore the clinical value of Bio‐C8 marker genes, we constructed a Bio‐C8 risk score based on the expression of CDC20, KIF20A and PTTG1 in Cohorts 2 and 3. Notably, the risk of LGG mortality increased as the risk score increased, and the gene expression levels of CDC20, KIF20A and PTTG1 gradually increased as the risk score increased in Cohort 2 and 3 (**Figure** **2**A,B). Patients classified as Grade 3 and IDH‐wild were also more enriched in the high‐risk group. Furthermore, our analysis revealed a significant difference in poor survival between patients with high‐ and low‐risk groups in LGG cohorts (Figure 2C,E). Importantly, a risk score based on the expression of CDC20, KIF20A and PTTG1 demonstrated the highest area under curve (AUC, Figure 2D,F) and remained an independent prognostic variable compared with indices of grade or IDH‐mutant status in Cox regression models (no collinearity with tolerance >0.1 and VIF < 10, Figure 2G,H). Immunohistochemistry was conducted on LGG primary tissue microarrays, and cases with higher levels of Bio‐C8 risk score based on CDC20, KIF20A and PTTG1 expressions showed lower overall survival (Figure 2I–K). These data demonstrated that stratification based on the expression levels of CDC20, KIF20A and PTTG1 was significantly associated with LGG prognosis.

### 2.3 CDC20^+^KIF20A^+^PTTG1^+^ Cell Subpopulation is Critical to Glioma Progression

**Figure 3:**
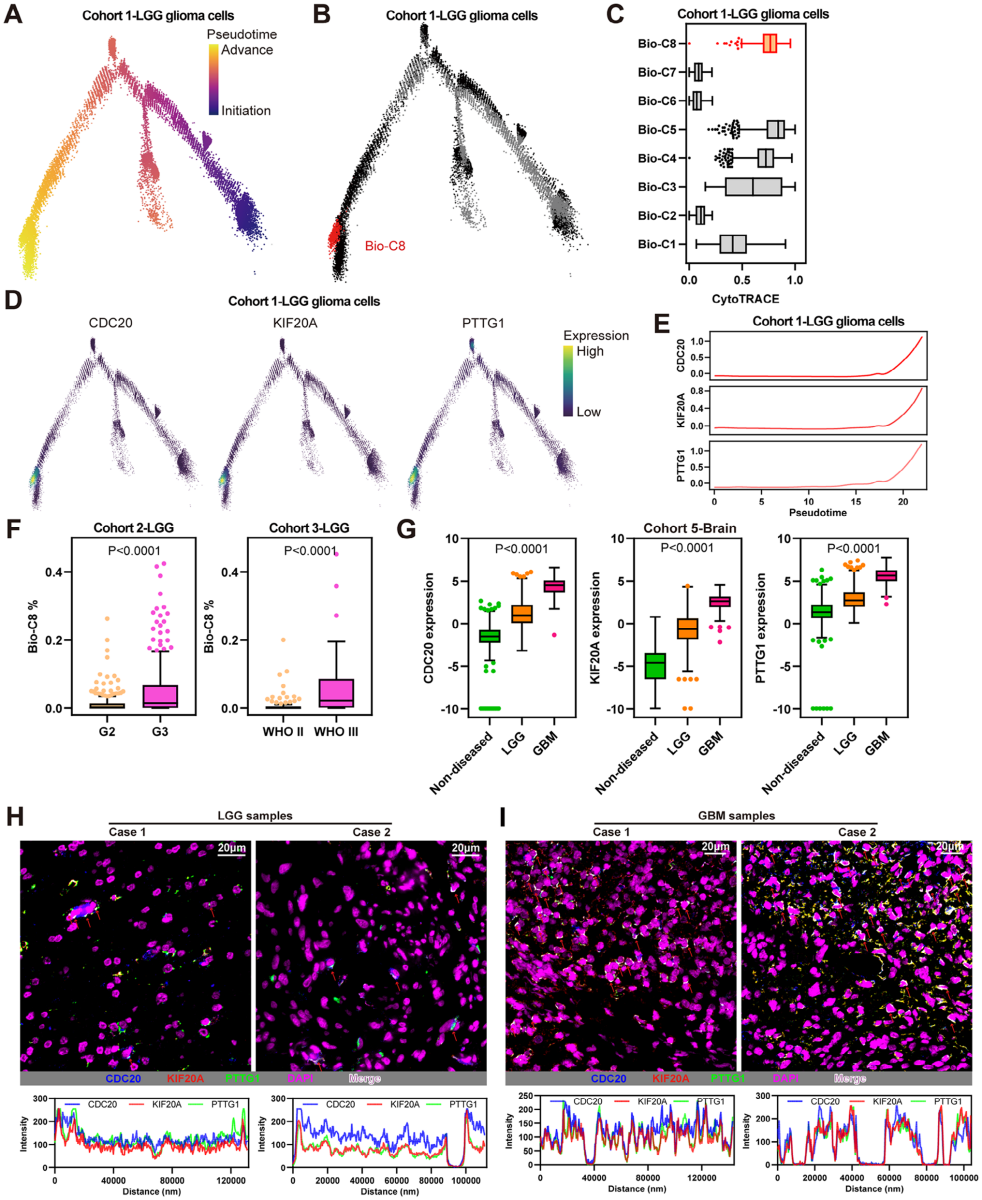
CDC20^+^KIF20A^+^PTTG1^+^ cell subpopulation is critical to glioma progression. A,B) Single‐cell trajectories of glioma cells colored by Pseudotime (A) and Bio‐C8 (B) in the cohort 1. C) Box plot showing the CytoTRACE levels grouped by eight biological‐clusters in the cohort 1. D) Single‐cell trajectories of glioma cells colored by expressions of CDC20, KIF20A and PTTG1 in the cohort 1. E) Expression pattern of CDC20, KIF20A and PTTG1 along pseudotime in the trajectory of glioma cells in the cohort 1. F) Box plot showing the Bio‐C8 proportions grouped by glioma initiation and progression in the cohort 2–3. G) Box plot showing the expression of CDC20, KIF20A and PTTG1 from the cohort 5 including healthy brain, LGG and GBM samples. H,I) Multiplex immunofluorescence images and corresponding analyses formed by CDC20, KIF20A and PTTG1 in LGG (H) and GBM (I) samples. Scale bar = 20 µm. Boxplots show median, quartiles, min and max.

To investigate why the CDC20^+^KIF20A^+^PTTG1^+^ cell (Bio‐C8) subpopulation is meaningfully associated with poor clinical outcomes in LGG, we analyzed the single‐cell trajectory of glioma cells in Cohort 1 using Monocle 2, this pseudotime trajectory recapitulated the progression of LGG glioma cells (**Figure** **3**A).^[^ ^19^ ^]^ According to the pseudotime model, the Bio‐C8 subpopulation is at the relative end position, which was confirmed with CytoTRACE algorithm (Figure 3B,C).^[^ ^20^ ^]^ We further observed that CDC20, KIF20A and PTTG1 are co‐expressed at the same position, consistent with the position of the Bio‐C8 (Figure 3D). Moreover, alongside which the degree of CDC20, KIF20A and PTTG1 expressions escalated at the advanced time point (Figure 3E). To further validate these results at the bulk level, we analyzed proportions of CDC20^+^KIF20A^+^PTTG1^+^ cell subpopulation from Figure 1E. Notably, the proportion in grade 3 LGGs was significantly greater than that of grade 2 LGGs (Figure 3F). It is well known that a subset of LGG will progress to GBM.^[^ ^1^ ^]^ Interestingly, the expression of CDC20, KIF20A and PTTG1 increased as the disease progressed in brain tissues from heathy, LGG and GBM (Figure 3G). Furthermore, abovementioned results showed that the CDC20^+^KIF20A^+^PTTG1^+^ cell subpopulation is associated with poor survival and advanced progression in LGG. Therefore, it is necessary to explore this unique cell subpopulation in GBM. We collected a high‐quality primary GBM scRNA‐Seq dataset from GEO. These cells processed and annotated refer to Cohort 1, as well as its previous report^[^ ^10^ ^]^ and different patients were clustered together in each cell type (Figure S2A–C, Supporting Information). Impressively, CDC20, KIF20A and PTTG1 were naturally and highly co‐expressed in GBM glioma cells (Figure S2D, Supporting Information). Further analysis showed that these three genes were mainly expressed in part of glioma cells rather than other glioma cells and non‐glioma cells (Figure S2E,F, Supporting Information). Finally, confocal fluorescence validated that these three genes were co‐expressed in validated glioma specimens, and that there were more CDC20^+^KIF20A^+^PTTG1^+^ cells in GBMs than in LGGs (Figure 3H,I). In conclusion, these findings suggest that the CDC20^+^KIF20A^+^PTTG1^+^ cell subpopulation is critical to glioma progression. These particular cells are likely as a subset of LGG will progress to GBM.

### 2.4 Knockdown of CDC20, KIF20A and PTTG1 Decreases Glioma Proliferation and Growth

**Figure 4:**
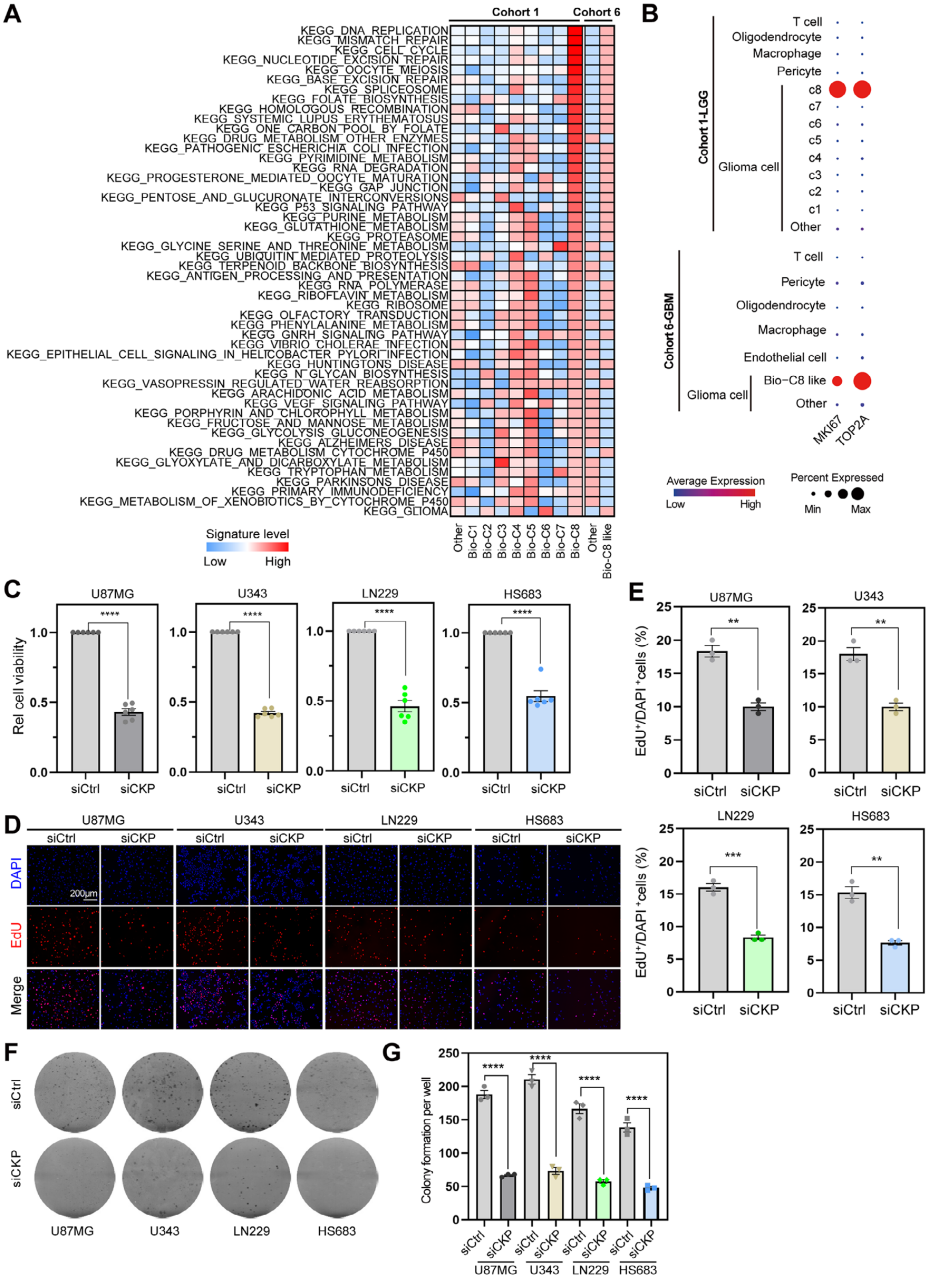
Knockdown of CDC20, KIF20A and PTTG1 decreases glioma proliferation and growth. A) Heatmaps showing correlation levels among each cell subpopulation and KEGG signature levels in glioma cells of cohort 1 and 6. B) Dot plot showing the expression of MKI67 and TOP2A in each cell type from the cohort 1 and 6. C) CCK8 assay showing cell proliferation of U87MG, U343, LN229 and HS683 cells after knockdown of CDC20, KIF20A and PTTG1. Data are shown as means ± SEM. D,E) The same group with (C) but for EdU experiments. Data are shown as means ± SEM. Scale bar = 200 µm. F,G) The same group with (C) but for colony formation experiments. Data are shown as means ± SEM. **P < 0.01, ***P < 0.001, ****P < 0.0001.

To further explore the molecular features of the CDC20^+^KIF20A^+^PTTG1^+^ cell subpopulation, we first used UCell to evaluate the molecular signature levels in single‐cell glioma cohorts (**Figure** **4**A).^[^ ^21^ ^]^ Cell cycle‐related, DNA repair/replication, ribosome and metabolism‐related signal pathways were enriched in the CDC20^+^KIF20A^+^PTTG1^+^ cell subpopulation of both LGG and GBM samples. Moreover, canonical oncogenic proliferation markers MKI67 and TOP2A were upregulated in the CDC20^+^KIF20A^+^PTTG1^+^ cell subpopulation of glioma samples (Figure 4B). Therefore, the CDC20^+^KIF20A^+^PTTG1^+^ cell subpopulation featured aberrant oncogenic molecular activity in glioma, suggesting that these cells are therapeutically vulnerable to glioma progression. Then, a series of phenotypic assays were performed to investigate whether CDC20, KIF20A and PTTG1 knockdown inhibits proliferation phenotypes in glioma cell lines. CDC20, KIF20A and PTTG1 combined knockdown inhibited cell viability (Figure 4C) and DNA replication (Figure 4D,E) in both LGG (LN229/HS683) and GBM (U87MG/U343) cancer cell lines. Constantly, combined knockdown decreased colony formation of glioma cell lines (Figure 4F,G). These data suggest that combined inhibition of the CDC20^+^KIF20A^+^PTTG1^+^ glioma cell subpopulation could provide novel strategies for molecular targeted therapy for glioma progression. These findings suggest that CDC20, KIF20A and PTTG1 promote glioma proliferation and growth.

### 2.5 CDC20^+^KIF20A^+^PTTG1^+^ Cell Subpopulation was Associated with Hypoxia in Glioma Progression

**Figure 5:**
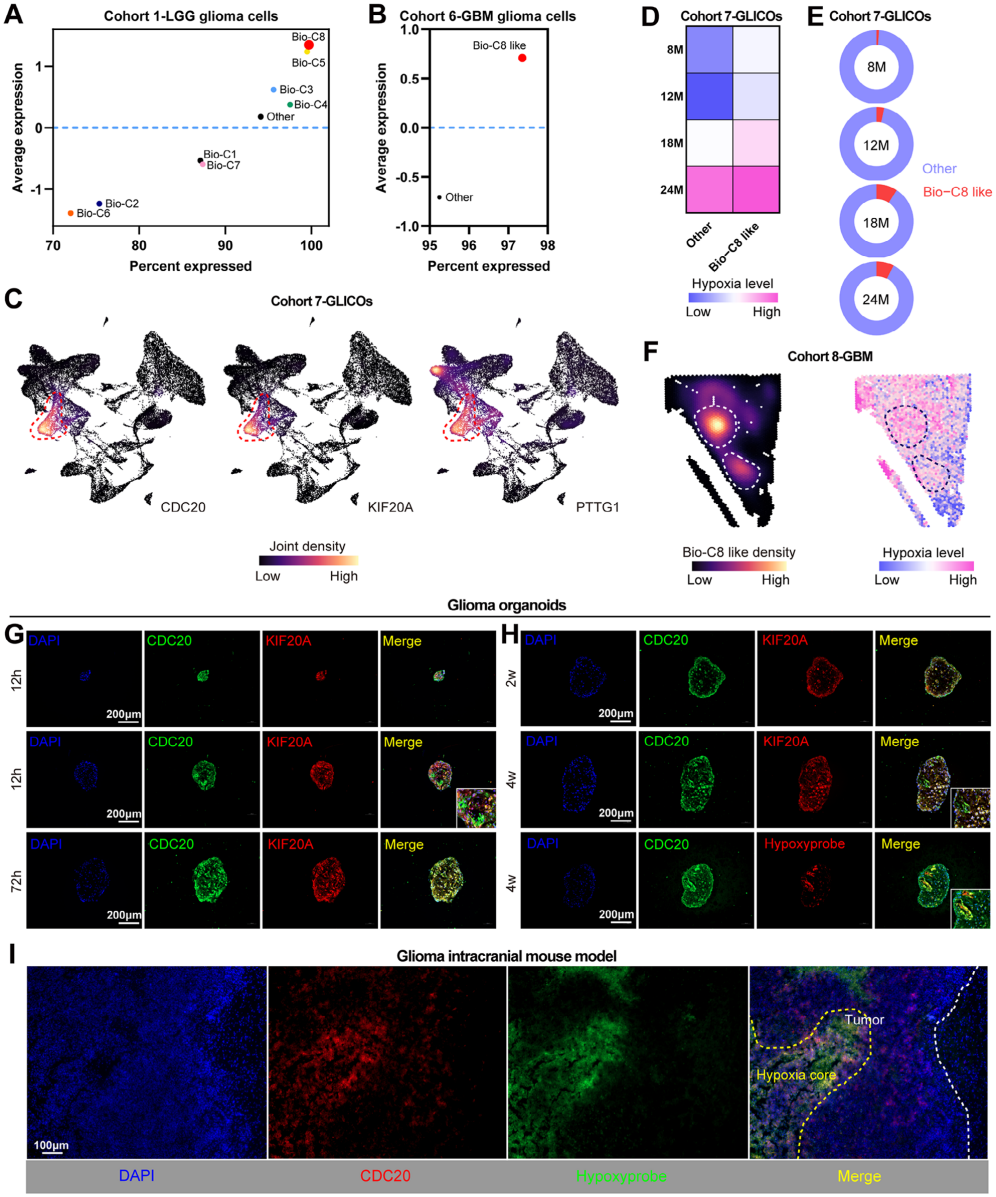
CDC20^+^KIF20A^+^PTTG1^+^ cell subpopulation was associated with hypoxia in glioma progression. A,B) Hypoxia levels of each biological‐clusters in glioma cells of cohort 1 and 6. The X‐axis shows proportion of hypoxia levels. The Y‐axis shows mean value of hypoxia levels. C) UMAP plots colored by expression density of CDC20, KIF20A and PTTG1 in the cohort 7 of the chronic hypoxia model. D) Heatmap showing the hypoxia level grouped by each cell population in time points from the cohort 7. E) Pie plots showing the proportion of each cell population in time points from the cohort 7. F) Spatial plots showing the CDC20^+^KIF20A^+^PTTG1^+^ cell subpopulation density and hypoxia level in cohort 8 GBM samples. G,H) Multiplex immunofluorescence images of CDC20 and KIF20A/hypoxyprobe in glioma PDOs under hypoxic (G) and normoxic (H) condition. Scale bar = 200 µm. I) Multiplex immunofluorescence images of CDC20 and hypoxyprobe in the long‐term glioma intracranial mouse model (4 weeks). Scale bar = 100 µm.

Hypoxia is prevalent in human tumors and has been identified a major key player driving aggressive phenotype of glioma, resulting in first‐line therapy resistance and recurrence.^[^ ^6^ ^]^ Moreover, the recent integrative spatial analysis of glioma indicated hypoxia appears to drive the layers that contains malignant cells in diverse states.^[^ ^7^ ^]^ Impressively, we used a 15‐gene signature that reflects hypoxia status appears to perform the best among other signatures to performed analysis and observed that the CDC20^+^KIF20A^+^PTTG1^+^ glioma cell subpopulation showed relative higher hypoxia level (**Figure** **5**A,B; Figure S3A, Supporting Information).^[^ ^22^, ^27^ ^]^ PDO of glioma is a fit pre‐clinical model of hypoxia.^[^ ^6^ ^]^ Thus, we used single‐cell profiling of the chronic hypoxia model based on long‐term cerebral organoids co‐cultured with patient derived glioma stem cells (GLICOs).^[^ ^11^ ^]^ Consistently, CDC20, KIF20A and PTTG1 were co‐expressed in glioma cells of GLICO (Figure 5C). CDC20^+^KIF20A^+^PTTG1^+^ glioma cell subpopulation remains relative higher hypoxia level than other glioma cells in each time point (Figure 5D), and increased during long‐term gliomagenesis (Figure 5E). MKI67 and TOP2A were also upregulated in the CDC20^+^KIF20A^+^PTTG1^+^ cell subpopulation of GLICO and gradually enhanced during long‐term gliomagenesis (Figure S3B, Supporting Information). Moreover, spatial atlas of GBM also showed that relative higher hypoxia level in spots of CDC20^+^KIF20A^+^PTTG1^+^ glioma cell subpopulation (Figure 5F). Importantly, we used GBM PDO under the hypoxic condition and observed that small‐sized PDO showed extensive activation of CDC20 and KIF20A in 12 h (Figure 5G). Relatively normal‐sized PDO showed activation of CDC20 but not KIF20A in hypoxia‐like core region at 12 h point, while both CDC20 and KIF20A were extensively activated at 72 h point (Figure 5G). When under the long‐term normoxic condition, hypoxia‐like core region and related CDC20 activation were not appeared at two weeks point, but appeared at four weeks point (Figure 5H). Furthermore, CDC20 was restrictively activated in the hypoxic area labeled by hypoxyprobe in the long‐term (4 weeks) glioma intracranial mouse model (Figure 5I). Hence, these results hint that CDC20^+^KIF20A^+^PTTG1^+^ high‐risk glioma cell subpopulation was specifically associated with hypoxia, which may drive this cell subpopulation predominantly via regulating its No.1 marker gene CDC20.

### 2.6 Targeting the CDC20^+^KIF20A^+^PTTG1^+^ Cell Subpopulation for Therapeutic Benefit in Glioma

**Figure 6:**
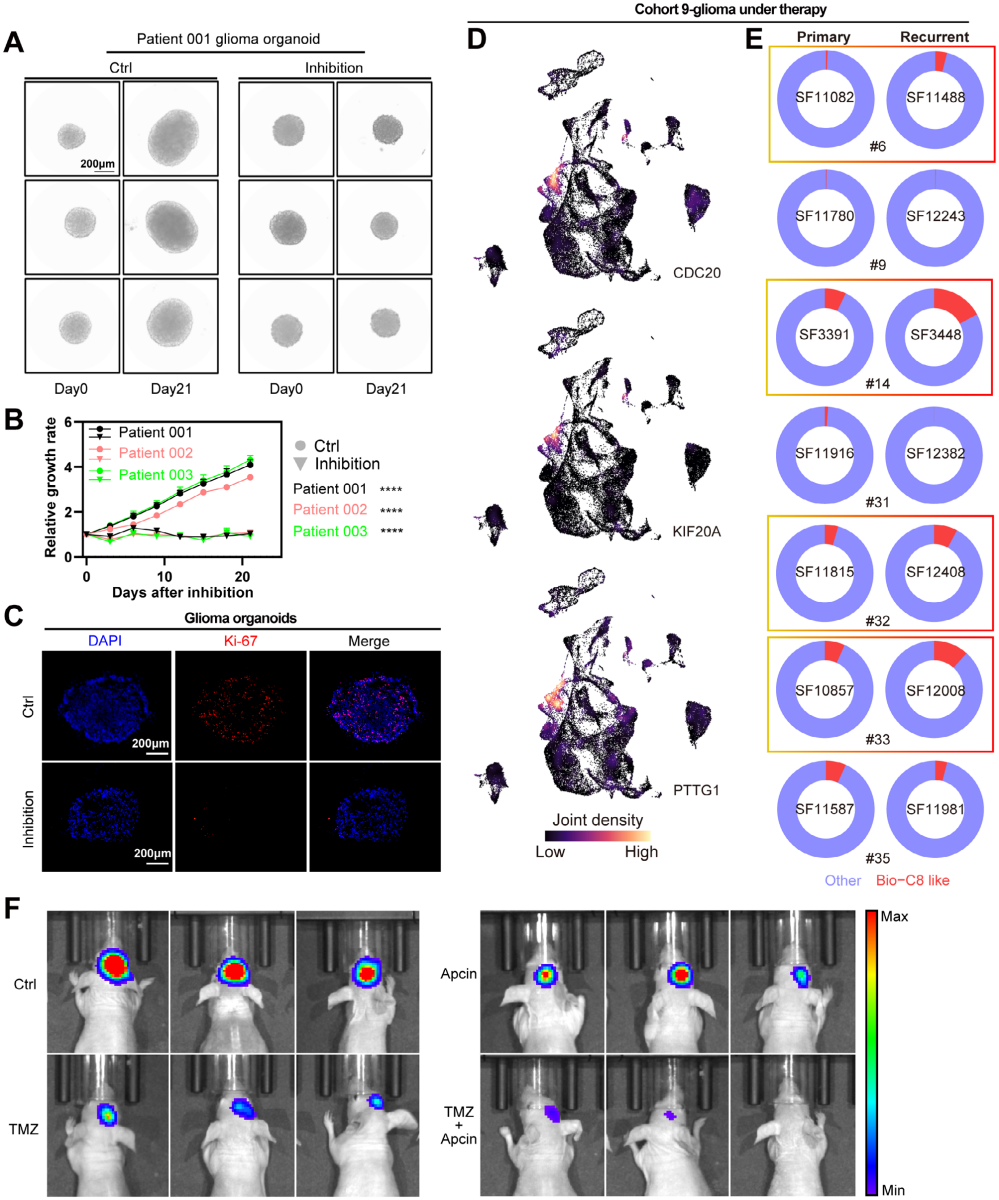
Targeting the CDC20^+^KIF20A^+^PTTG1^+^ cell subpopulation for therapeutic benefit in glioma. A,B) Growth curve of glioma PDOs after combined inhibition of CDC20 and KIF20A. Data are shown as means ± SEM. Scale bars = 200 µm, ****P < 0.0001. C) Immunofluorescence showing the Ki67^+^/DAPI^+^ cells (%) of glioma PDOs after combined inhibition of CDC20 and KIF20A. Scale bars = 200 µm. D) UMAP plots colored by expression density of CDC20, KIF20A and PTTG1 in the cohort 9 of gliomas under therapy. E) Pie plots showing the proportion of each cell population in glioma cells grouped by primary and recurrent from the cohort 9. F) Representative bioluminescence images of tumor bearing mice after combined treatment of TMZ (20 mg kg^−1^) and CDC20 inhibitor Apcin (15 mg kg^−1^) every other day for 2 weeks.

Finally, we combined CDC20 and KIF20A related inhibitors Apcin and Paprotrain to performed therapy experiments in glioma PDOs. Combined treatment significantly attenuated organoid growth and proliferation in glioma PDOs (**Figure** **6**A,B; Figure S4A, Supporting Information). After inhibition, Ki67‐positive areas were decreased and concentrated in locations with higher CDC20 expression (Figure 6C; Figure S4B–D, Supporting Information). Furthermore, we used glioma single‐cell atlas of cases treated only with standard‐of‐care therapy (TMZ, radiation and surgical resection, Figure S4E,F, Supporting Information).^[^ ^13^ ^]^ We analyzed glioma cells of seven‐matched recurrent and primary cases that contain survival and recurrence information. CDC20, KIF20A and PTTG1 remained co‐expressed in glioma cells (Figure 6D), and this cell subpopulation was increased in four recurrence glioma cases under therapy (Figure 6E). TMZ is the first‐line chemotherapeutic for glioma treatment, inducing DNA damage.^[^ ^28^ ^]^ Interestingly, spatial atlas of GBM showed that relative higher DNA repair signaling in spots of CDC20^+^KIF20A^+^PTTG1^+^ glioma cell subpopulation (Figure S4G, Supporting Information). DNA repair pathways may be used by these glioma cells for creating TMZ resistance. Hence, these results suggest that CDC20^+^KIF20A^+^PTTG1^+^ glioma cell subpopulation is potential target in effective TMZ‐sensitizing therapies. Consistently, glioma cell derived xenograft models showed that inhibition of this cell subpopulation enhanced TMZ treatment efficiency (Figure 6F).

## 3 Discussion

The coordinated activity of highly specialized cell types is essential for the function of the mammalian brain.^[^ ^29^ ^]^ Brain tumors are a typical example of heterogeneous tumors and one of the most lethal human malignancies.^[^ ^15^ ^]^ Emerging evidence suggests that intra‐tumoral heterogeneity among tumor and non‐tumor cells and their interactions within the TME are critical to each aspect of tumor biology.^[^ ^30^ ^]^ Advances in high‐throughput scRNA‐seq analysis have provided an opportunity to illuminate the genetic properties of brain tumors and reveal heterogeneity within the TME. To explore how expression states vary among different cells, an NMF algorithm was developed and used in the microenvironment‐related studies. For instance, Welch et al. used NMF to learn a low‐dimensional space in human and mouse brain cell data.^[^ ^29^ ^]^ Puram et al. used NMF to identify coherent sets of genes that were preferentially co‐expressed by subsets of tumor cells in head and neck cancer.^[^ ^30^ ^]^ Furthermore, Mathewson et al. used NMF to robustly identify sub‐clusters in T cells of gliomas.^[^ ^31^ ^]^ In our study, we used NMF to learned and defined intra‐tumor expression programs that consist of co‐expressed genes in each LGGs. A series of intra‐tumor expression programs were dissected followed by biological‐cluster classification. Bio‐C8 LGG cases was associated with poorer clinical outcomes, suggesting potential correlation with glioma development. Thus, we focused on this biological‐cluster and further investigated it. Besides, we also noted that Bio‐C3 displayed most improved survival time in LGGs, suggesting that this subgroup finds relatively hard to develop GBM.

To obtain sign genes of Bio‐C8 subpopulation more precision, we designed a unique method based on NMF‐defined genes and bulk level datasets. Consistently, CDC20, KIF20A and PTTG1 were the top three genes in both two LGG cohorts at the bulk level. CDC20 is a well‐known cell division cycle gene, as it controls the correct segregation of chromosomes during mitosis.^[^ ^32^ ^]^ Mitotic defects activate the spindle‐assembly checkpoint, which inhibits the anaphase‐promoting complex co‐activator CDC20 to induce a prolonged cell cycle arrest.^[^ ^33^ ^]^ KIF20A is a kinesin family member. Loss of KIF20A function does not cause defects in cell division or apoptosis but induces early cell cycle exit and early differentiation of neurons.^[^ ^34^ ^]^ PTTG1 is a homolog of the yeast Securin protein gene and a regulator of sister chromatid separation.^[^ ^35^ ^]^ The gene product contains unique motifs that are necessary for its tumorigenic activities. Importantly, Cox regression models revealed that risk scores based on the expression of CDC20, KIF20A and PTTG1 were independent prognostic variables than indices of tumor grade or IDH‐mutant status. Our further immunohistochemistry experiments confirmed that patients with higher expression levels of CDC20, KIF20A and PTTG1 had shorter overall survival, which means that the expression of CDC20, KIF20A and PTTG1 is an independent prognostic factor for patients with LGGs. Further analysis revealed that the CDC20^+^KIF20A^+^PTTG1^+^ cell subpopulation is associated with poor survival and advanced progression in LGG. This subgroup is naturally present at high grades and shows more CDC20^+^KIF20A^+^PTTG1^+^ cells than LGG, suggesting a critical role in glioma progression. Enrichment analysis also suggested the potential oncogenic proliferation of this cell subtype. Interestingly, a recent study highlighted that cell proliferation may be a driving force behind primary glioma progression before treatment.^[^ ^36^ ^]^ To verify its primary role, we constructed in vitro cell proliferation and ex vivo organoid models and experiments, showing that it plays an important role in the proliferation of gliomas. The inhibition of CDC20 and KIF20A showed significantly inhibited proliferation in organoid models, suggesting that this subpopulation is a potential therapeutic target. However, M.‐J. Tsang and I. M. Cheeseman revealed a close correlation between the relative CDC20 proteoform ratio, mitotic arrest duration and anti‐mitotic drug sensitivity,^[^ ^33^ ^]^ which means that Apcin should be avoided in combination with anti‐mitotic drugs. Due to the absence of the inhibitor for PTTG1, we were unable to evaluate the effect of the combination of three genes’ inhibitors.

Hypoxia and its key regulator hypoxia‐inducible factor 1 (HIF‐1) play key roles in tumor growth, malignant progression and treatment resistance.^[^ ^37^ ^]^ HIF‐1 activates the transcription of many genes that are involved in changes in gene expression in angiogenesis, metabolism, cell proliferation, metastasis and other cellular processes. Interestingly, Min Shi et al. found that CDC20‐mediated PHD3 ubiquitination prevents the interaction between VHL and HIF‐1α in liver cancer, thereby preventing it from VHL‐mediated degradation and leading to increased stability and activity of the HIF‐1α protein.^[^ ^38^ ^]^ CDC20^+^KIF20A^+^PTTG1^+^ cell subpopulation showed the highest hypoxia status among other cells in glioma according to the single‐cell atlas. Our experiments further hint that this cell subpopulation, in particular its marker gene CDC20, was higher in the hypoxia core region of glioma PDOs, suggesting that CDC20 plays a vital role in the glioma hypoxic microenvironment. Interestingly, previous studies showed that glioma PDOs allowed to grow larger developed hypoxia gradients, which is a hallmark of GBM.^[^ ^23^, ^39^ ^]^ Thus, these findings suggest that there probably exists a loop mechanism between CDC20^+^KIF20A^+^PTTG1^+^ cell subpopulation and hypoxia core activation, leading to the glioma progression.

Collectively, our comprehensive analyses and experiments revealed a novel hypoxia‐associated high‐risk cell subpopulation involved in glioma progression. This study further provides a personalized prognostic method and may contribute to precision cancer therapies. This glioma cell subpopulation is therapeutically vulnerable to glioma progression and may play important roles in glioma standard‐of‐care therapy, such as TMZ treatment. Our findings provide comprehensive insights into therapeutic strategies for glioma progression, highlighting the promise of preventing early‐stage glioma progressing to advanced disease.

## 4 Experimental Section

### Data Resources

Cohort 1 comprised four LGGs cases were obtained from the surgical specimen archives of the Department of Neurosurgery, Beijing Tiantan Hospital, Capital Medical University. Then, a single‐cell RNA‐seq examination was performed by experimental personnel in the laboratory of SEEKGENE. Cohort 2 of TCGA LGG cases was downloaded from the Genomic Data Commons Data Portal (https://portal.gdc.cancer.gov/). Cohort 3 of CGGA LGG cases was downloaded from the Chinese Glioma Genome Atlas (http://www.cgga.org.cn/index.jsp).^[^ ^9^ ^]^ Cohort 4 glioma tissue microarrays were purchased from Shanghai Outdo Biotech Company (Cat No. HBraG159Su01). Cohort 5 of the GTEx healthy and TCGA glioma brain integrate dataset was obtained from UCSC Xena (http://xena.ucsc.edu/). Cohort 6 of GBM single‐cell data was obtained from GSE103224 (https://www.ncbi.nlm.nih.gov/gds).^[^ ^10^ ^]^ Cohort 7 of glioma chronic hypoxia model single‐cell data was obtained from GSE210736.^[^ ^11^ ^]^ Cohort 8 of GBM spatial data was obtained from GSE194329.^[^ ^12^ ^]^ Cohort 9 of glioma standard‐of‐care therapy single‐cell data was obtained from GSE174554.^[^ ^13^ ^]^ Multiplex immunofluorescence and PDOs samples were obtained from the surgical specimen archives of the Department of Neurosurgery and Emergency Medicine, Jiangnan University Medical Center (Wuxi No.2 People's Hospital), and were acquired and processed under procedures approved by the institutional review boards of the Jiangnan University Medical Center (Wuxi No.2 People's Hospital). All tissue samples were collected in compliance with informed consent policy.

### Single‐Cell Annotation

Seurat package of R software was used to perform single cell cluster and annotation.^[^ ^14^ ^]^ We annotated the cell type represented by each cluster by considering the known cell type markers: GFAP and PTPRZ1 (glioma cell); MOG (oligodendrocyte); RGS5 (pericyte); PECAM1 (endothelial cell); CD3D and CD3E (T cell); CD68 (macrophage).

### Single‐Cell CNV Calling

The chromosomal copy number variations profile of single cells was inferred using the inferCNV R package.^[^ ^15^ ^]^ Immune cells and stromal cells were used as a reference to define a baseline of normal karyotype. The clonal large‐scale chromosomal copy number variations of malignant cells were estimated by sorting the analyzed genes by their chromosomal location and applying a moving average to the relative expression values.

### Deciphering Intra‐Tumor Expression Programs and Biological‐Clusters

To explore underlying intra‐tumor expression signatures of glioma cell in LGGs, it was applied non‐negative factorization to glioma cells in cohort 1 patients using NMF R package refer to the previous single‐cell study.^[^ ^16^ ^]^ First, expression counts were normalized for each tumor and replaced all negative values in the expression matrix by zero. Next, the top 10 ranked NMF programs in each tumor sample were dissected. Then, the top 100 genes were extracted with the highest weight for each NMF program. Finally, it was used a clustering analysis to all NMF program based on the pair‐wised Jaccard index calculated similar to the previous description^[^ ^17^ ^]^: Jaccard index = A∩B/A∪B, where A and B represent top 100 genes of two NMF programs. Biological‐clusters were defined shared by multiple tumors based on hierarchical clustering and manually annotated.

### Evaluation of Cell Populations at the Bulk Level

Deconvolution module of BayesPrism was used to infer a series of single‐cell subpopulation in bulk RNA‐seq datasets.^[^ ^18^ ^]^ A Bayesian method to predict cellular composition and gene expression in individual cell types from cohort 2 and 3, using cohort 1 scRNA‐seq data as prior information.

### Survival Analysis

Two grouped survival analyses of biological‐clusters proportion at the bulk level were performed as previously described^[^ ^17^ ^]^: cases were divided into high and low proportion groups for each biological‐cluster, differences in P value were examined in the overall survival of the groups according to a Kaplan–Meier survival analysis, the value yielding the lowest log‐rank p‐values from the 10th to 90th percentiles of the samples was selected. Risk score of Bio‐C8 used Z‐score transferred mean values of CDC20, KIF20A and PTTG1. Kaplan–Meier, ROC curve and COX analyses were used GraphPad Prism 10 and R software 4.3.0.

### Pseudotime Analysis

Monocle2 was used to perform pseudotime inference in glioma cells of the cohort 1.^[^ ^19^ ^]^ CytoTRACE algorithm was used to reconstruct cellular differentiation trajectories in glioma cells of the cohort 1.^[^ ^20^ ^]^

### Enrichment Analysis

Ucell package of R software was used to calculate a series of hallmark signature levels in glioma cells of the cohort 1 and 6.^[^ ^21^ ^]^ Hallmark signature genesets were obtained from the Molecular Signatures Database (MSigDB) (https://www.gsea‐msigdb.org/gsea/msigdb/collections.jsp). A 15‐gene expression signature (ACOT7, ADM, ALDOA, CDKN3, ENO1, LDHA, MIF, MRPS17, NDRG1, P4HA1, PGAM1, SLC2A1, TPI1, TUBB6 and VEGFA) that has been shown to perform the best when classifying hypoxia status was selected to evaluate in glioma cells of the cohort 1 and 7.^[^ ^22^ ^]^

### Cell Lines and Culture Conditions

The human glioma cell lines U87MG, U343, A172 and HS683 were obtained from the Cell Bank of Type Culture Collection of the Chinese Academy of Sciences (Shanghai, China) and regularly authenticated by short tandem repeat analysis and tested for the absence of Mycoplasma contamination. All cells were cultured in DMEM (Hyclon, Logan, UT, USA) with 10% fetal bovine serum (FBS; Invitrogen, Carlsbad, CA), 1% penicillin and streptomycin (P/S; Hyclon). These cells were characterized by Genewiz, Inc. (China) using short tandem repeat markers and were confirmed to be mycoplasma‐free.

### PDO Creation, Culture and Biobanking

Fresh surgically resected glioma tissue was placed in sterile phosphate buffered saline and taken immediately to the Department of Pathology to confirm a preliminary diagnosis of gliomas by the attending neuropathologist. The tissue was transferred to a sterile glass dish with H+GPSA medium containing Hibernate A, 1X GlutaMax (Thermo Fisher Scientific), 1X PenStrep (Thermo Fisher Scientific) and 1X Amphotericin B (Thermo Fisher Scientific) for dissection under a stereomicroscope (Zeiss).^[^ ^23^ ^]^ 200–500 µm diameter pieces of glioma tissue were distributed in ultra‐low attachment 6‐well culture plates (Corning) with 4 mL of glioma organoid medium containing 50% DMEM:F12 (Thermo Fisher Scientific), 50% Neurobasal (Thermo Fisher Scientific), 1X GlutaMax (Thermo Fisher Scientific), 1X NEAAs (Thermo Fisher Scientific), 1X PenStrep (Thermo Fisher Scientific), 1X N2 supplement (Thermo Fisher Scientific), 1X B27 minus vitamin A supplement (Thermo Fisher Scientific), 1×2‐mercaptoethanol (Thermo Fisher Scientific) and 2.5 µg ml^−1^ human insulin (Sigma) per well and placed on an orbital shaker rotating at 120 rpm within a 37 °C, 5% CO_2_, 20% oxygen(Figure 5) or 5% oxygen (all other figure) and 90% humidity sterile incubator.^[^ ^24^ ^]^ Roughly 75% of the medium was changed every 48 h by tilting the plates at a 45° angle and aspirating the medium above the sunken organoids. To freeze, organoids were grouped up to six per well in Short‐Term Glioma Organoid Medium containing the ROCK inhibitor Y‐27632 (final conc. 10 µM, Stem Cell Technologies 72 302) for 1 h. Next, DMSO (10% v/v) was added and organoids and media were transferred to a cryotube. They were incubated at 4 °C for 15 min, frozen at –80 °C, and stored long‐term in a cryogenic freezer under liquid nitrogen vapor. All organoids presented in the manuscript were cultured for a minimum of four weeks before analysis. For the sphere growth assay, photographs of tumor spheres were taken at the indicated time points and the sphere area was measured. The sphere growth was expressed as the ratio of the area at different time points compared to the area at day 1.

### Cell Growth Assay and Colony Formation Assay

For cell growth assays, a total of 1000 cells were seeded into 96‐well plates and monitored by Cell Counting Kit‐8 according to the manufacturer's protocol at the indicated time points. For colony formation assays, 1000 cells were seeded into six‐well plates and maintained in a complete medium for 14 days. The colonies were fixed with 4% paraformaldehyde (PFA) (Sangon, Shanghai, China) and stained with 0.1% crystal violet (Sangon, China), the number of colonies was counted using an inverted microscope.

### EdU Labeling Assay

EdU labeling assay was performed to examine the rate of DNA replication. In brief, cells were cultured in 24‐well plate for 36 h followed by an incubation of 10 ≤ µm EdU (KeyGen Biotech, China) for 2–3 h and fixation with 4% PFA. The staining procedure was performed according to the manufacturer's instructions for the kFluor594 Click‐iT EdU Kit (KeyGen Biotech, China). After staining, the coverslips were mounted with Gelmount containing Hoechst 33342 (Sigma) and photographed under an Axio Imager Z2 Fluorescence Microscopy (Carl Zeiss).

### Immunohistochemistry Staining

Immunohistochemistry staining was conducted by Wuhan servicebio technology company (Wuhan, China). Tissue slides were incubated with antibodies CDC20 (1:200, Proteintech Cat# 10252‐1‐AP, RRID: AB_2 229 016), KIF20A (1:400, Proteintech Cat# 67190‐1‐Ig, RRID: AB_2 882 485), PTTG1 (1:200, Proteintech Cat# 18040‐1‐AP, RRID: AB_2 173 410). DAB staining intensity was analyzed using Aipathwell.

### Double Immunofluorescence Staining

Briefly, the fixed cells or tissue sections were premobilized and blocked with 0.3% Triton X‑100 or 3% normal goat serum in 0.01 M phosphate‐buffered saline (PBS) for 30 min at room temperature (RT), followed with an overnight incubation of indicated antibodies CDC20 (1:200), KIF20A (1:400), PTTG1 (1:200) and Ki67(1:1000, BD Biosciences Cat# 550 609, RRID: AB_393 778) at 4 °C. On the following day, the cells or sections were incubated with fluorescein isothiocyanate‑conjugated goat anti‑rabbit and TRITC‑conjugated goat anti‑mouse (1:100; Jackson ImmunoResearch Laboratories, Inc., West Grove, PA, USA) antibodies. Cell nuclei were counterstained with Hoechst 33 342 (Invitrogen). The sections were washed, mounted and examined using the Axio Imager Z2 Fluorescence Microscopy (Carl Zeiss). Primary antiserum omission and normal mouse and goat serum controls were used to confirm the specificity of the immunofluorescent labeling.

### Multiplexed Immunofluorescence Staining

Multiplex staining of glioma tissue and organoids was performed using PANO 4‐plex IHC kit (Panovue, Cat# 10 216 100 100) according to manufacturer's instructions. Briefly, tissue slides were first deparaffinized and then incubated sequentially with primary antibodies CDC20 (1:200), KIF20A (1:400) and PTTG1 (1:200) followed by horseradish peroxidase‐conjugated secondary antibody incubation and tyramide signal amplification. The slides were microwave heat‐treated after each tyramide signal amplification operation. Nuclei were stained with DAPI after all the antigens above had been labeled. The immunofluorescence images were captured via microscopy (Carl Zeiss LSM880, Zeiss, Germany), which captures the fluorescent spectra at 20 nm wavelength intervals from 420 to 720 nm with identical exposure time. Multilayer images were imported to ZEN for quantitative image analysis. The quantities of various cell populations were expressed as the number of stained cells per square millimeter and further as the percentage of positively stained cells.

### Intracranial Mouse Model and Hypoxic Probe

All animal studies were approved by the Experimental Animal Ethics Committee of Jiangnan University. Male BALB/c nude mice (4 weeks old, n = 5/group) and C57BL/6 (4 weeks old, n = 4) mice were purchased from GemPharmatech and maintained in micro isolator cages. Male BALB/c nude mice with lateral abdominal tumors were randomly divided into 4 groups, which were given saline, Temozolomide (TMZ, HY‐17364, MCE), Apcin (HY‐110287, MCE) and TMZ+Apcin once every other day for 14 days. The hypoxic probe was purchased from Hypoxyprobe, Inc. (HypoxyprobeTM‐1 Plus Kit, HP2‐100Kit). The intraperitoneal injection was conducted in C57BL/6 intracranial mouse model after 4 weeks followed by the Assay Instructions.

### Ethics Approval and Consent to Participate

Glioma samples were collected from Jiangnan University Medical Center (Wuxi No.2 People's Hospital) with informed consent as the previous study described. This project was approved by the Clinical Research Ethics Committees of Jiangnan University Medical Center (Wuxi No.2 People's Hospital, 2023‐Y‐70). All the animal experiment was approved by the Experimental Animal Ethics Committee of Jiangnan University (JN. No20240515b0300830[248]).

### Statistical Analysis

Figures were designed, analyzed and visualized by GraphPad Prism 10 and R software 4.3.0. Patients’ survival has been analyzed as described in the above corresponding section. Mann–Whitney/T test was used in the two‐group comparison and the Kruskal–Wallis test was used in the three‐group comparison using GraphPad Prism 10. All reported p‐values were two‐sided. A P value less than 0.05 was regarded as statistically significant. Sample size information was described in the above corresponding section. Data presentation and P values of experimental data were shown in the corresponding figure legends.

## Conflict of Interest

The authors declare no conflict of interest.

## Author Contributions

Q.W., X.W., and J. Z. contributed equally to this work. X.L., K.C., Qing W., and J.C. supervised this project and mentored the participants. J.Z. and J.C. supervised scRNA‐seq related LGG samples’ collection and processed; J.Z., W.W., and J.C. performed scRNA‐seq related LGG samples’ collection and processed; Quan W., X.W., and K.C. designed LGG scRNA‐seq examination; Quan W. and K.C. designed and performed the bioinformatics analyses and visualization; Z.G. and Q.L. provided comments and support for bioinformatics analysis. Quan W., X.W., Z.G., Qing W., K.C., and X.L. designed the wet‐lab experiments; Quan W., X.W., H.T., and K.C. performed clinical validation; Quan W., X.W., C.S., Y.C., and Z.L. performed in vitro function experiments; X.W., Qing W., and X.L. supervised ex vivo PDO samples collected and Biobank collection; Quan W., X.W., C.S., and Z.L. performed ex vivo PDO models’ construction and experiments; Quan W. performed in vivo models’ construction and experiments. All authors discussed the results. Quan W. and K.C. wrote the manuscript. Qing W., X.W., K.C., and X.L. critically revised the manuscript.

## Supporting information



Supporting Information

## Data Availability

The data that support the findings of this study are available from the corresponding author upon reasonable request.
