# Supplementary material for: The Hypoxia‐Associated High‐Risk Cell Subpopulation Distinctly Enhances the Progression of Glioma
Source: Adv Sci (Weinh). 2025 Mar 6;12(17):2416231. doi: 10.1002/advs.202416231 (PMC12061283; doi:10.1002/advs.202416231)
Supplement: Supplementary file 1 — Supporting Information [file ADVS-12-2416231-s001.docx]

**The hypoxia-associated high-risk cell subpopulation distinctly** **enhances the progression of glioma**


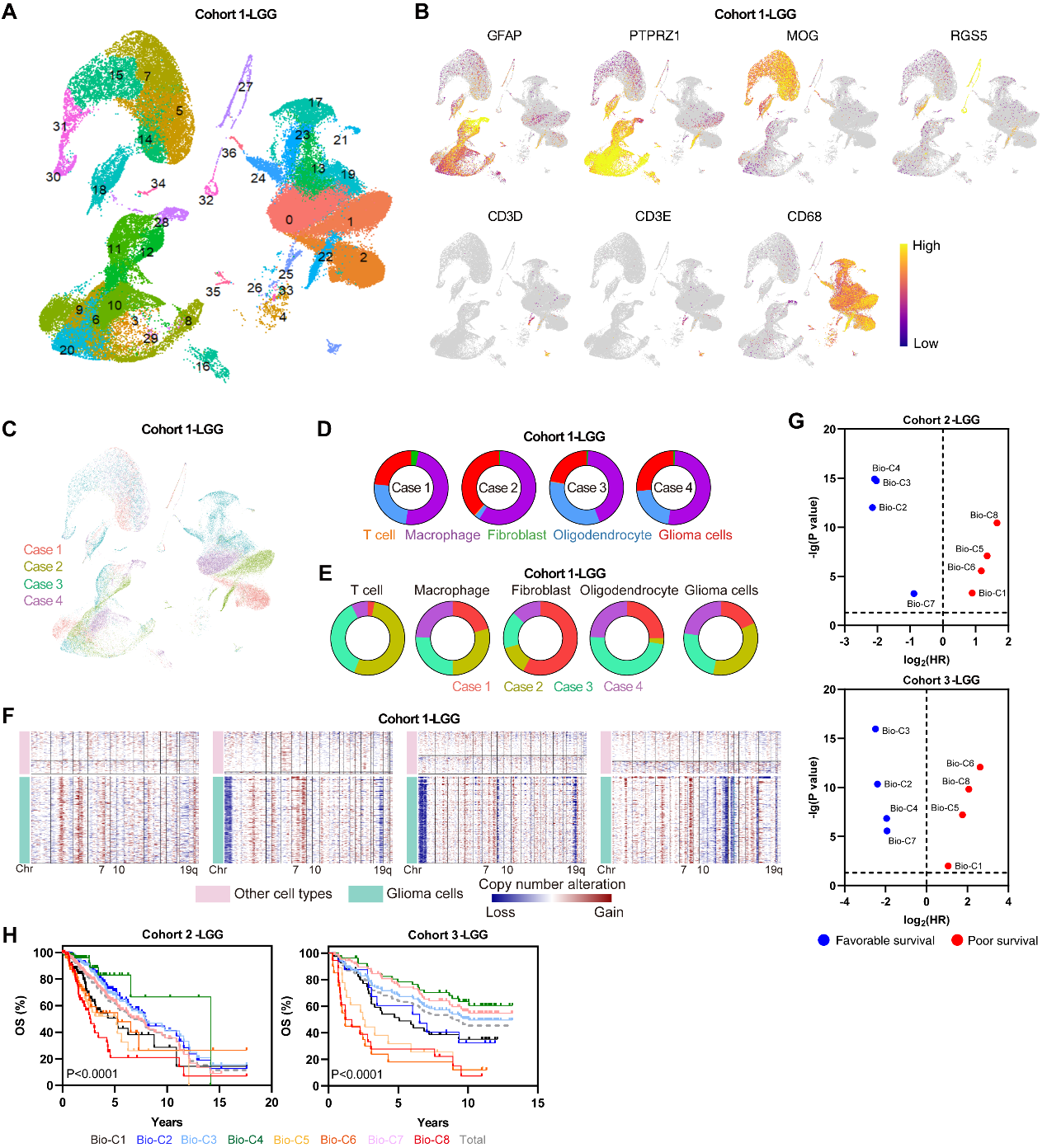


**Figure. S1 Related to Figure. 1.**

**A**. UMAP plots colored by clusters.

**B**. UMAP plots colored by expression of each marker genes from the cohort 1.

**C**. UMAP plots colored by cases from the cohort 1.

**D**. Pie plots colored by cell types and grouped by cases from cohort 1.

**E**. Pie plots colored by cases and grouped by cell types from cohort 1.

**F**. Heatmaps colored by copy number alteration levels grouped by cell types in each case from cohort 1.

**G**. Kaplan–Meier survival analyses of OS for eight biological-clusters in cohort 2 and 3. The X-axis shows the log_2_ transformed hazard ratio (HR) values. The Y-axis shows the -lg transformed P values.

**H**. Kaplan–Meier curves showing the OS of each higher biological-cluster group in cohort 2 and 3.


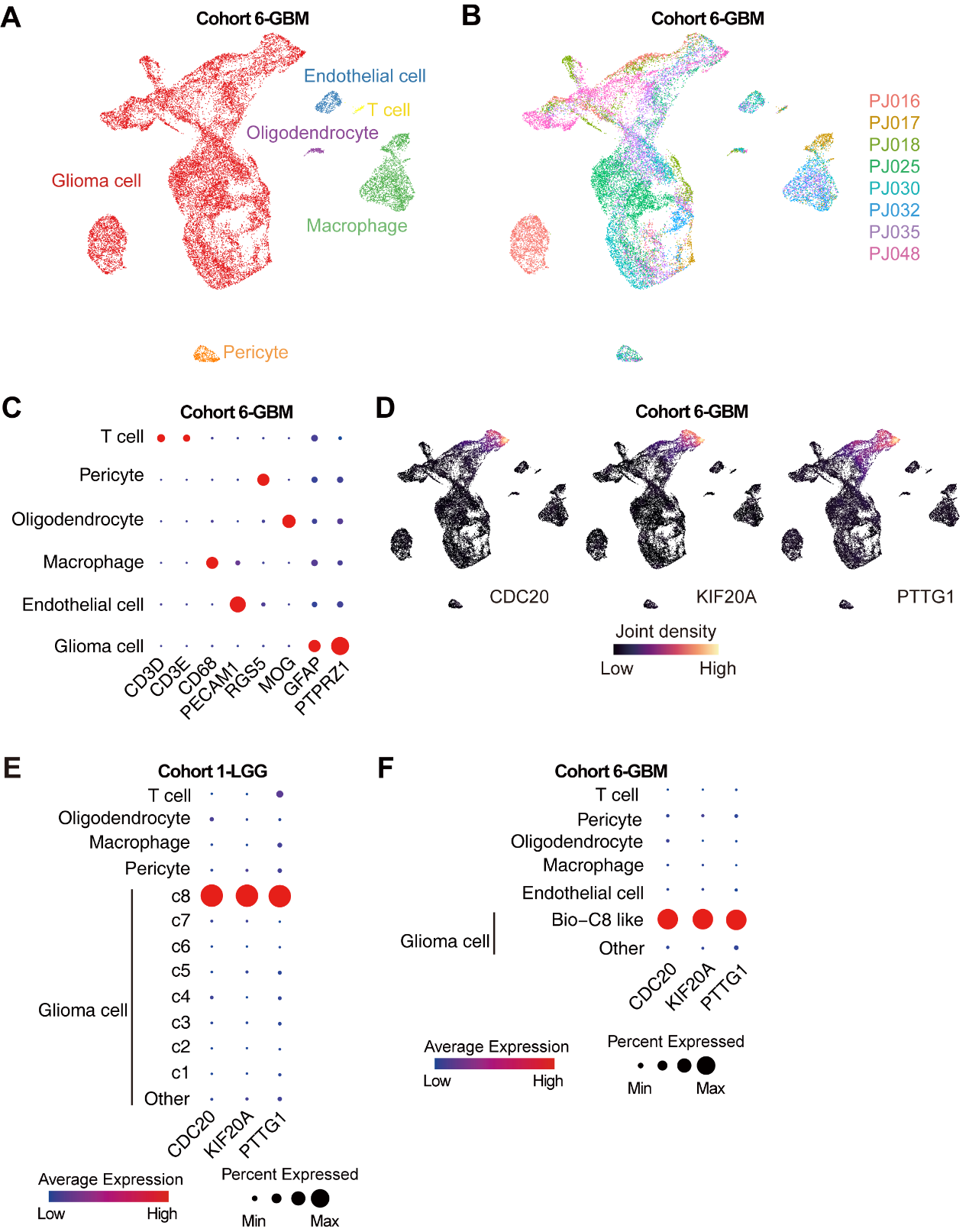


**Figure. S2 Related to Figure. 3.**

**A-B**. UMAP plots colored by cell types (**A**) and cases (**B**) from the cohort 6 of GBMs.

**C**. Dot plot showing marker gene expressions in each cell type from the cohort 6.

**D**. UMAP plots colored by expression density of CDC20, KIF20A and PTTG1 in the cohort 6.

**E-F**. Dot plot showing the expression of CDC20, KIF20A and PTTG1 in each cell type from the cohort 1 and 6.


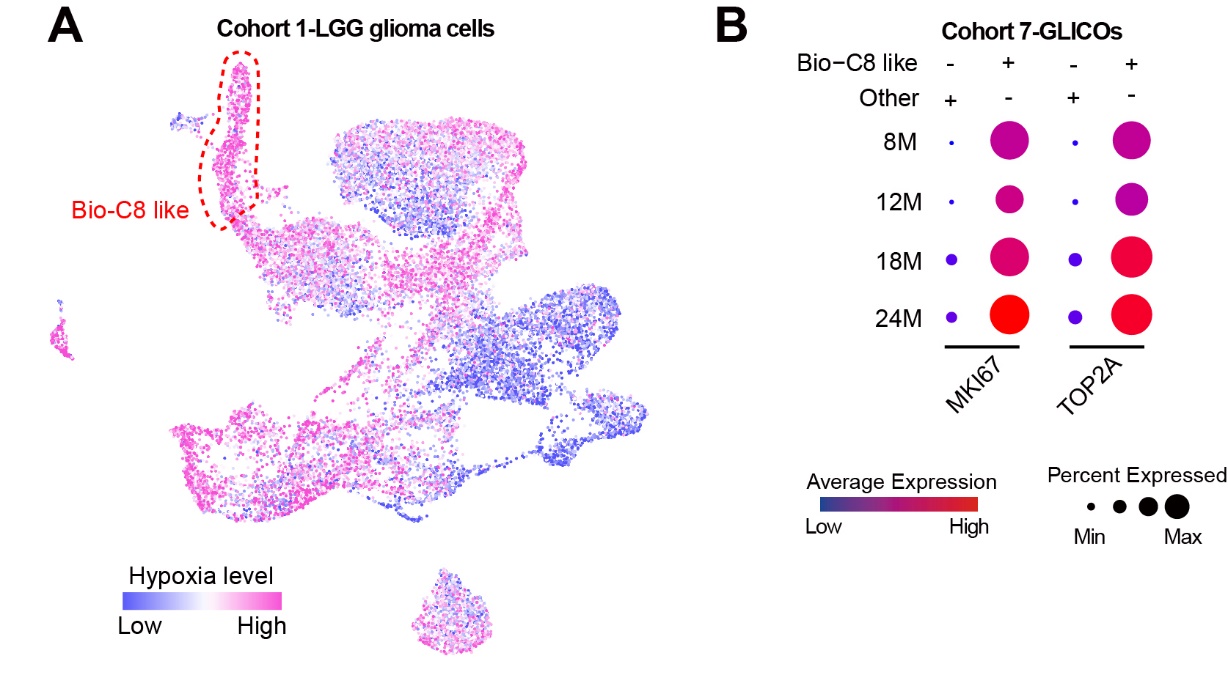


**Figure. S3 Related to Figure. 5.**

**A**. UMAP plots showing the hypoxia level in glioma cells from the cohort 1.

**B**. Dot plot showing the expression of MKI67 and TOP2A of each cell population in time points from the cohort 7.


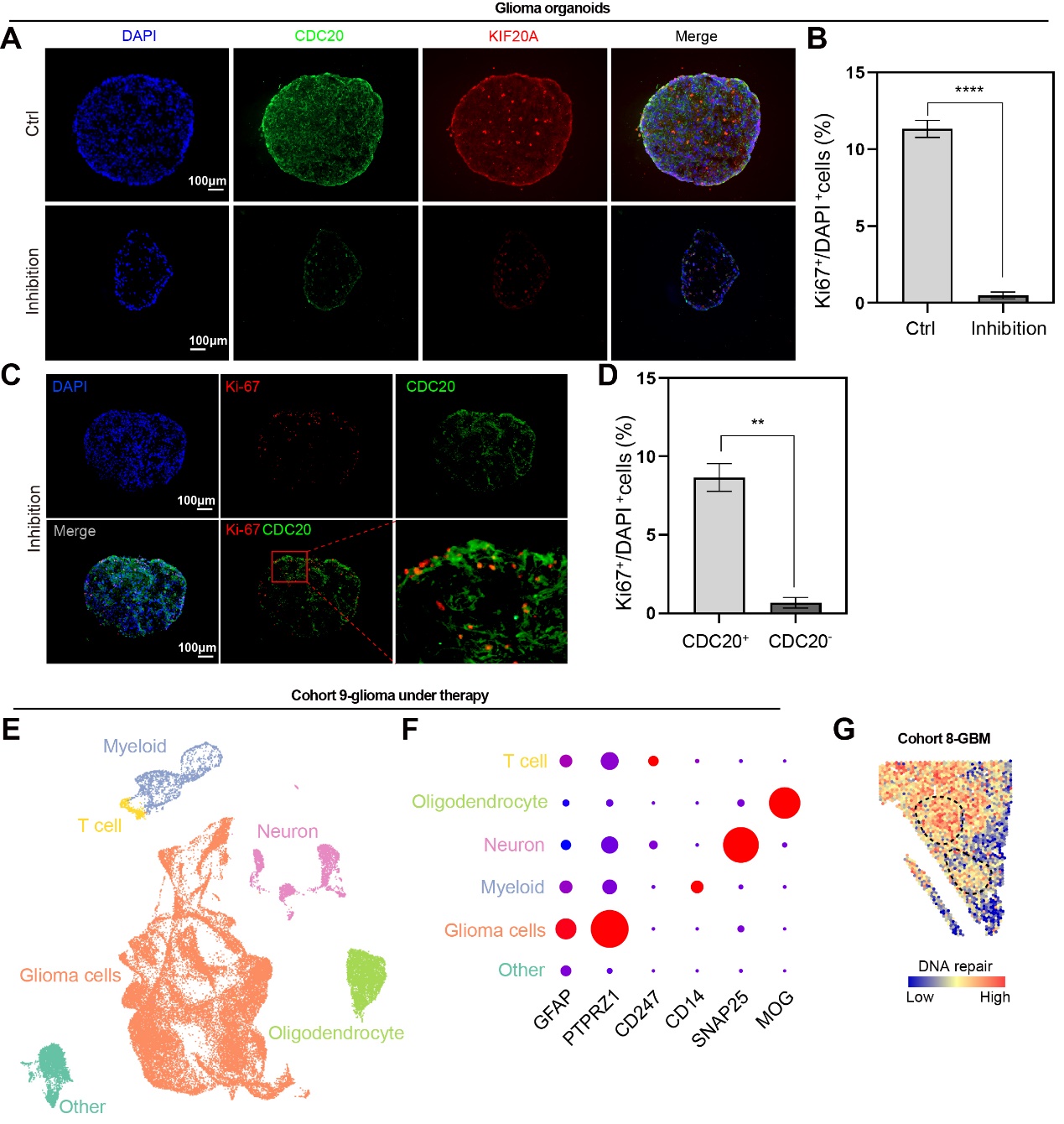


**Figure. S4 Related to Figure. 6.**

**A**. Immunofluorescence showing the expression of CDC20 and KIF20A of glioma PDO after combined inhibition of CDC20 and KIF20A. Scale bars = 100 µm.

**B**. Bar plot showing the Ki67^+^/DAPI^+^ cells (%) of glioma PDO after combined inhibition of CDC20 and KIF20A, related to **Figure. 6C**. Data are shown as means ± SEM.

**C-D**. Immunofluorescence and bar plot showing the Ki67^+^and CDC20^+^ cells of glioma PDO after combined inhibition of CDC20 and KIF20A. Scale bars = 100 µm. Data are shown as means ± SEM.

**E**. UMAP plots colored by cell types from the cohort 9.

**F**. Dot plot showing marker gene expressions in each cell type from the cohort 9.

**G**. Spatial plot showing the DNA repair signaling level in the cohort 8 sample.

**P<0.01, ****P<0.0001.
